# Spatial Dynamics of Student Conflicts in Spanish Higher Education: Gender and Academic Discipline Differences in Campus Spaces

**DOI:** 10.3390/bs16050681

**Published:** 2026-04-29

**Authors:** Gloria Rojas-Ruiz, Ángel Custodio Mingorance-Estrada, Inmaculada Alemany-Arrebola

**Affiliations:** 1 Department of Didactics and School Organization, University of Granada, 18012 Granada, Spain; amingoe@ugr.es; 2 Department of Psychological Development and Education, Universidad de Granada, 18012 Granada, Spain; alemany@ugr.es

**Keywords:** interpersonal conflicts among students, campus space, gender differences, academic disciplines, higher education, student well-being

## Abstract

Universities function as social systems in which physical, structural and symbolic dimensions shape students’ relationships and well-being. This study explored the university spaces in which students perceive and manage interpersonal conflicts, analysing differences by gender and academic discipline. A sample of 1262 students completed the University Conflict Questionnaire (UCQ). The three-dimensional structure was validated using exploratory (EFA) and confirmatory factor analysis (CFA). Reliability was assessed using Cronbach’s alpha. Inferential analysis included Welch’s *t*-test and ANOVA to compare gender and year group, estimating the effect using Hedges’ g. Finally, a MANOVA was conducted to test for interaction effects. The results indicate that conflicts are most frequently addressed in shared and interactive areas, whilst institutional and study spaces are less prone to conflict. Classrooms were key contexts for both the emergence and resolution of conflicts, underscoring the role of lecturers in fostering inclusion. Women reported fewer conflicts in institutional spaces, and differences related to discipline were modest. The results highlight the influence of structural conditions and the need for institutional strategies that incorporate students’ perspectives and training in mediation. These findings highlight the importance of considering university spaces in the design of strategies to promote coexistence and student well-being.

## 1. Introduction

The university, as both an educational and social institution, constitutes a space where diverse human, cultural and structural dynamics intersect. Therefore, the analysis of higher education environments is key to understanding how interpersonal relationships, learning processes and academic life are shaped. It is essential that students perceive their university as a safe, inclusive and respectful environment. This perception directly influences their academic development and well-being (Atasanova & Papen, 2025; Brint et al., 2024; Misischia, 2022). Given that the physical environment plays a fundamental role in mental health and well-being (Ross et al., 2022), students who perceive their institution as trustworthy are more likely to engage actively in learning, interact positively with peers and staff, and demonstrate higher levels of academic self-efficacy (Clausen et al., 2025).

Analysing conflicts in university spaces therefore provides valuable insights into how social interactions, institutional culture and spatial design influence educational and emotional outcomes. This issue became particularly salient following the COVID-19 pandemic, when homes were transformed into virtual learning environments and universities worldwide had to rethink their pedagogical models to re-establish their campuses as spaces for learning, social interaction and academic life (Danvers & Wells, 2025; Thompson et al., 2025). In the wake of this post-pandemic context, the literature has increasingly incorporated the role of digital environments in the university experience. Wu et al. (2025) demonstrate that social anxiety and the fear of missing out (FoMO) influence student relationships, broadening the understanding of conflict to include hybrid dynamics that combine face-to-face and digital interaction.

As Lozano (2020) argues, understanding the origins, evolution and spatial dimensions of potential conflicts is necessary for their prevention and proper management. The structural configuration of the university is a key variable that enables conflicts to be identified and comprehensively understood (Munuera & Martínez, 2024).

From a theoretical perspective, the analysis of conflict in higher education requires moving beyond individual-level explanations and considering the role of context. Sociocultural approaches (Vygotsky, 1978) emphasise that learning and development are fundamentally shaped through social interaction, while ecological models of human development (Bronfenbrenner, 1979) highlight the influence of multiple environmental systems on individual behaviour. These perspectives suggest that student conflict is not an isolated phenomenon. Instead, it is a situated process that develops within structured contexts.

In this sense, the concept of spatial dynamics provides a useful analytical lens. In this study, spatial dynamics are understood as the interplay between physical, social and institutional characteristics of university spaces that shape the emergence, experience and management of interpersonal conflicts. This perspective allows for a more comprehensive understanding of how campus design, patterns of interaction and institutional structures influence students’ relational experiences.

Furthermore, gender constitutes a fundamental axis in the use of university space, since, according to various studies, women face specific perceptions and challenges in relation to the environment, which differentiates their experiences from those of men (Gargiulo et al., 2020; Huang et al., 2022; Ramos & Pérez, 2025). Research has consistently shown that women perceive higher levels of insecurity in certain campus contexts, particularly in relation to sexual harassment and other forms of violence (Huang et al., 2022; Prada-Trigo et al., 2025), affecting mobility and the use of space, and influencing participation and social interaction (Jaskulska et al., 2026). Therefore, gender differences must be interpreted in relation to perceived safety, social norms and the institution’s responsiveness.

Finally, academic discipline also plays a significant role in shaping interpersonal relationships. Mavor et al. (2025) show that the degree programme students are enrolled in influences the way they relate to their peers and the institution, affecting both their academic engagement and their educational experience. Recent studies show that well-being and motivation vary across disciplines, suggesting the existence of distinct academic cultures that may influence the emergence and management of conflicts (Laitinen et al., 2025). Hernández et al. (2022) highlight that the degree programmes students are enrolled in generate and reproduce distinct practices within social spaces, shaping their behaviours and identities from their first year of enrolment.

Taken together, these contributions allow us to conceptualise the university as a complex public space where physical, social and institutional dimensions converge. Student conflict emerges as a situated and relational phenomenon, shaped by campus design, interaction dynamics, social identities and academic structures. From this perspective, analysing the spaces in which conflicts arise and are managed allows for a better understanding of the conditions that foster coexistence, well-being and the academic development of the student body.

This study was conducted at the University of Granada, one of Spain’s oldest public universities and the only one with campuses on two continents: Europe and Africa. The participating students were enrolled in undergraduate programmes related to the field of education.

The aim of this study is to identify the campus spaces where students perceive interpersonal conflicts to arise and how they manage these in everyday interactions, considering gender and academic discipline as key variables. A deeper understanding of these dimensions is essential for guiding educational policies and promoting institutional cultures that foster reflection, dialogue and inclusion, thereby contributing to students’ well-being and academic development.

## 2. The University as a Public Space: An Analysis from the Perspective of Space and Coexistence

Physical space is not a neutral factor in educational processes; it is an active component that shapes inclusion, coexistence and well-being, functioning as profoundly social environments that generate attachment and reinforce a sense of belonging to the community (Bart-Plange et al., 2025; Van Kessel et al., 2025). In this regard, Wong (2024) proposes the concept of ‘spatial belonging’ to explain how this sense of belonging is shaped through the physical, relational, digital and structural dimensions of the university space, moving beyond reductionist views that confined it to the individual psychological level. Therefore, from an educational perspective, they function as learning environments that can facilitate or hinder social interaction, the acquisition of knowledge, and the development of personal and collective skills (Guo et al., 2023).

Dong et al. (2023) argue that university public spaces should be understood as interactive environments that foster learning, the exchange of ideas, social interaction and leisure. Similarly, Velez and Jessup (2022) emphasise that viewing the university as a living space requires analysing it not only from an institutional perspective, but also as an environment that promotes quality education. This holistic approach recognises the interdependence between academic content, the physical environment and social interactions as a central element of students’ comprehensive education.

Thus, the university constitutes an educational and social ecosystem in which cultural, economic and generational dimensions intersect (Arias, 2018). Domínguez et al. (2022) and Kassab et al. (2024) highlight that learning environments directly influence students’ well-being and academic performance, enhancing their ability to acquire knowledge and resolve conflicts. This strengthens coexistence and peace within educational institutions (Chávez & Norzagaray, 2021).

From a perspective more focused on learning environments, the recent literature has emphasised the role of the classroom as a key space in shaping social interactions. Makaremi et al. (2024) note that the physical and social environment of the classroom influences students’ well-being, whilst Konstantidinis (2024) identifies four major factors that affect this well-being: teaching staff, classroom atmosphere, course design and interpersonal relationships as determinants of student well-being. In this regard, the classroom is not only a space for the transmission of knowledge, but also a social context in which conflicts can arise and be managed.

Referring to recent studies by Guo et al. (2023) and Solís et al. (2024), university common spaces are essential for students’ physical and mental well-being, yet many campuses still fail to fully meet these needs. Although there is a growing debate on how to create healthy and restorative environments, systematic research examining how spatial characteristics influence student behaviour remains limited.

Historically, learning spaces in higher education have received little academic attention, and few studies have incorporated students’ own perspectives (Huang et al., 2022; Perry & Wiewel, 2015; Temple, 2008). For a long time, it was believed that university education took place independently of the physical environment, although this perspective has evolved. Dong et al. (2023) note a significant increase in studies focusing on students’ perceptions of university spaces from 2018 onwards, with the aim of improving campus design and optimising users’ emotional and cognitive experiences. These developments reflect a paradigm shift towards people-centred planning that prioritises well-being and meaningful interaction.

In this context, Thompson et al. (2025), building on Hitch et al. (2014), analyse the meaning of the campus through four interconnected dimensions: the space to do (skills and abilities); space to be (self-awareness and social roles); space to become (growth and development); and space to belong (connections with people, places and communities).

Much of the research on perceptions of the campus has focused on gender differences in terms of safety. Studies consistently show that women face greater insecurity and restrictions on their freedom of movement, particularly in relation to sexual harassment (Huang et al., 2022; Prada-Trigo et al., 2025; Ramos & Pérez, 2025).

Academic discipline also shapes interpersonal relationships and conflict management within university spaces. Student well-being varies depending on the demands of their degree programme, course structure, teaching practices, year of study and personal factors (Li, 2024; Szepe & Meszanos, 2024). Hernández et al. (2022) explain that students become part of disciplinary communities that cultivate their own traditions, beliefs and styles of interaction. These communities distinguish one programme of study from another and contribute to students’ sense of institutional belonging. These dynamics begin in the first year, a critical stage for adaptation and interaction within diverse groups and environments, both on and off campus.

These theoretical contributions demonstrate that the university space is not merely a physical environment, but a structural and symbolic component of higher education. Its design, its uses and the ways in which it is appropriated directly shape students’ personal, academic and social development. Understanding the university as a public space therefore requires recognising it as an environment where citizenship is built, relationships are forged, conflict resolution is learnt, and democratic values are strengthened.

This work forms part of a line of research that views the university as a public space for education, socialisation and coexistence. Its contribution lies in empirically examining how conflicts are distributed and managed across different areas of the campus, incorporating two particularly relevant axes of differentiation in the recent literature: gender, due to its relationship with safety and the differential appropriation of space; and academic discipline, due to its role in shaping identities, relationships and cultures of interaction.

With this in mind, the aim of this study is to analyse the influence of the university environment on the emergence and management of student conflicts, and to determine whether there are distinct patterns based on gender and academic discipline.

For all these reasons, the research questions guiding this study are:

1. In which university spaces do students perceive conflicts to arise?
2. In which spaces do students typically manage these conflicts?
3. What differences arise in terms of gender and academic discipline?

Based on these questions, the following hypotheses are proposed:

**H1.**  *Perceptions of the source of conflict are concentrated in environments characterised by high levels of social interaction and academic activity, with a lower incidence in institutional areas.*

**H2.**  *The distribution of conflict resolution spaces differs from that of the conflict’s source, with a preference for settings that facilitate dialogue or mediation over purely administrative ones.*

**H3.**  *Gender and academic discipline act as differentiating variables, generating significant variations in the assessment and use of campus environments in the face of conflict.*

## 3. Methodology

This research is framed within a retrospective ex post facto design with a single group and multiple response measures (Ato et al., 2021). This approach is appropriate when the independent variables (gender, degree programme and campus) cannot be manipulated by the researchers, as they represent characteristics inherent to the subjects or situations that have already occurred.

### 3.1. Participants

The study was conducted across the three campuses of the University of Granada, one of Spain’s oldest universities and the only one with campuses on two different continents: Europe and Africa.

The sample comprises 1262 students from the Faculties of Education Sciences, belonging to the aforementioned campuses. The description of the participants shows a mean age of 21.18 years (SD = 3.56), ranging from 17 to 46 years. In terms of gender, 200 are men (20.4%) and 1015 (79.6%) are women, reflecting the feminisation of degree programmes related to the field of education. According to the degree programme they are studying, 468 are Early Years Education students (36.6%); 415 are Primary Education students (32.4%); 234 are Social Education students (18.3%) and 163 are studying Pedagogy (12.7%).

Participants were selected using a non-probabilistic, purposive or convenience sampling method, choosing subjects who met the criteria appropriate for the study’s purposes: being enrolled in an undergraduate degree programme related to the field of education at the University of Granada. No additional exclusion criteria were established, as the aim was to obtain a representative sample of students studying in this academic field.

Although the research was conducted at a single university, the study included participants from all three campuses of the University of Granada, which allowed for the capture of internal variability within the same institutional framework. This feature is particularly relevant, as the university has a unique configuration within the Spanish context due to its presence on two continents, Europe and Africa. Consequently, the design is not limited to a single-campus framework, but is situated within an internally diverse university context, suitable for examining how the spatial and institutional conditions of the campuses relate to the perception and management of interpersonal conflicts among students.

### 3.2. Instrument

For this study, the ‘Questionnaire on Conflict at University (CCU)’ (Rojas et al., 2024a) was administered, comprising eight sections (Table 1).

For this research, which forms part of a wider study, sections 6 (“Areas where conflicts arise”) and 7 (“Areas where conflicts are resolved”) are analysed (Table 2).

### 3.3. Procedure

To administer the questionnaire, contact was made with the lecturers responsible for the courses, both in person and via electronic means. During this exchange, the objectives of the research were explained in detail, and the most suitable date and time for administering the questionnaire during academic sessions were agreed upon. The questionnaire was administered in person and took approximately fifteen minutes to complete.

The research was conducted in strict compliance with the ethical principles set out in the Declaration of Helsinki, ensuring at all times the voluntary participation of the subjects, anonymity, the confidentiality of the information collected, and the use of the data exclusively for scientific purposes. Furthermore, students were informed of the anonymous nature of the questionnaire and of their right to decline to participate or to withdraw at any stage of the study, without this having any repercussions.

### 3.4. Data Analysis

The statistical analysis of the data was carried out hierarchically, beginning with the psychometric validation of the instruments corresponding to Blocks 6 (sources of conflict) and 7 (conflict management) using Exploratory Factor Analysis (EFA). The results of the EFA confirmed the existence of three distinct dimensions in each block, which allowed for a precise operationalisation of the dependent variables by calculating the mean scores of the items for each factor. It is important to note that, although the data present a hierarchical structure (students nested within programmes and campuses), a fixed-effects analysis was chosen instead of multilevel modelling. This decision is based on the primary objective of the research: identifying transversal differences by gender and academic discipline, rather than studying the specific variance attributable to the centres. Additionally, the use of Welch’s test and the calculation of effect sizes guarantee a parsimonious and robust interpretation of the results, which is appropriate for the scope of this study.

The reliability of Blocks 6 and 7 was also verified using Cronbach’s alpha, yielding satisfactory values for the study sample (N = 1262).

In the inferential phase, Welch’s *t*-test and analysis of variance (ANOVA) were applied to compare the variables of gender and academic year independently. The choice of Welch’s *t*-test was justified following confirmation of a breach of the assumption of homoscedasticity (Levene’s test, *p* < 0.05) and an imbalance in group sizes. Furthermore, parametric tests were employed, taking into account the sample size, even in the absence of strict normality (Schmidt & Finan, 2018; Tsagris & Pandis, 2021). Effect sizes were also calculated to assess the magnitude of the differences. It should be noted that, although participants were grouped by year group and degree programme, traditional statistical procedures were maintained because the main objective of the study was to examine the relationships between variables at the individual level, rather than to estimate group effects; therefore, the use of multilevel models was not considered necessary for the purposes of this research.

To assess the practical relevance of the findings, the effect size was estimated using Hedges’ g, selected for its precision in samples of unequal sizes compared to Cohen’s d. Finally, a multivariate analysis of variance (MANOVA) was conducted to test for possible interaction effects between gender and academic year, ensuring a comprehensive interpretation of the model’s combined variance and controlling the Type I error rate, with the significance level at *p* < 0.05 for all analyses.

## 4. Results

### 4.1. Preliminary Analyses of the CCU

Firstly, the reliability and validity of the instruments were analysed. Regarding block 6, ‘Spaces where conflicts arise’, the internal consistency analysis showed an adequate consistency index of α = 0.789. After examining item–total correlations, it was observed that item 1, “Classes”, had the lowest correlation (0.162); however, it was retained in the final instrument for reasons of content validity and substantive relevance. With regard to the Exploratory Factor Analysis (EFA), conducted using the principal components method with Varimax rotation, it revealed an underlying three-dimensional structure explaining 58.49% of the total variance of the phenomenon. The model fit was confirmed by a KMO index of 0.782 and a significant Bartlett’s sphericity test (χ^2^ = 5026.99; *p* < 0.001), which justifies the factorisation of the matrix. The first component, “Institutional Spaces” (23.92% of variance), groups the following items: Deans’ Offices, Vice-Deans’ Offices, Guidance Services, Offices and Administration; these are places where conflict tends to have a marked hierarchy, with factor loadings of up to 0.893. The second factor, “Workspaces” (21.29%), groups the following items: Library, Study Rooms, Photocopier and Other Spaces. These are places intended for study or work. The third factor, “Spaces for informal interaction and leisure” (13.28%), comprises the items Classes, Corridors and Cafeteria; these areas of informal or collective socialising indicate spaces for diffuse interaction.

With regard to block 7, “Spaces where conflicts are resolved”, the scale shows good internal consistency with an α = 0.805, ensuring that the 12 items measure the construct of spaces where conflicts are resolved in a stable and coherent manner. AFE revealed an underlying three-dimensional structure which, in this case, explains 61.25% of the total variance of the phenomenon. The model fit was optimal, with a KMO index of 0.817 and a significant Bartlett’s sphericity test (χ^2^ = 5154.51; *p* < 0.001), confirming the robustness of the factor structure. The first factor, termed “Interaction and Leisure Spaces” (25.85% of the variance), groups the items: Corridors, Photocopier, Library, Off-campus spaces and Study rooms; these results suggest that much of the interaction occurs in settings of spontaneous social interaction. The second factor, “Institutional Spaces” (25.26%), comprises the items: Dean’s Offices, Guidance Services, Vice-Dean’s Offices, Offices and Administration, representing the institutional and hierarchical route to conflict resolution. Finally, the third factor, “Work and Relational Space” (10.14%), appears independently, comprising the items Classes and Cafeteria with a loading of 0.871, which identifies these spaces as a setting with distinct resolution dynamics.

From a psychometric perspective, the reconfiguration of the factor loadings in blocks 6 and 7 demonstrates that university spaces are not static variables, but functional constructs that transform according to the stage of the conflict (genesis vs. resolution). The greater robustness of the resolution model, evidenced by a higher KMO (0.817) and an increase in explained variance (61.25% compared to 58.49% in the original), suggests that the subjects possess a more defined and clear cognitive structure regarding where to manage conflicts than regarding where they occur.

### 4.2. Descriptive and Inferential Analyses

This study analysed the university spaces where interpersonal conflicts most frequently arise and are managed, using a Likert-type rating scale, where 1 = Never and 4 = Always. Table 3 shows the results obtained, which allow us to establish a clear map of the places where students most frequently identify situations of interpersonal conflict, both on and off campus and where they manage them.

With regard to where university conflicts arise (Table 3), the data indicate that the ‘Classrooms’ setting (Item 1) is the predominant setting for conflicts, with the highest mean score (M = 2.76; 62.2%). The high frequency here suggests that conflict is intrinsically linked to academic activity; it is the only item that exceeds the 60% threshold, suggesting a higher concentration of conflicts in this setting.

Other spaces that may be critical for generating conflict are those outside the classroom, namely ‘Corridors’ (34.8%) and ‘Canteen’ (28.5%), where there is less supervision but a high level of social interaction. These ‘transit’ spaces exhibit moderate levels of conflict. As these are areas of informal interaction, the conflicts that arise tend to be interpersonal in nature and less linked to academic matters. “Spaces outside the campus” (33.7%) suggest a “delocalisation of conflict”, likely fuelled by interactions on social media, messaging groups or leisure environments shared by students. However, it is in this category that high variability is found (SD = 0.938), indicating greater polarisation or a lack of consensus among participants regarding the frequency of conflicts in this space. Finally, there are spaces where conflict is almost non-existent: these are the Vice-Dean’s Offices (M = 1.42; 4.8%) and Dean’s Offices (M = 1.42; 7.4%), which show the lowest means (M = 1.42). The skewness of these data (close to 1.5) indicates that respect for the hierarchical structure and the sporadic nature of visits to these offices inhibit the emergence of disputes. Finally, the ‘Library’ (9.1%) and the ‘Study Rooms’ (13.9%) also show low levels, as the social norm of silence acts as a natural preventive mechanism.

With regard to conflict resolution scenarios (Table 4), the descriptive results confirm that the classroom is the primary setting for conflict management. This item has the highest mean (M = 2.64) and low dispersion (SD = 0.78), standing out particularly for its skewness index close to zero (0.004) and with a slightly lower percentage than where they originate (56.8%). This value indicates a normal distribution of responses, reflecting a unanimous consensus within the university community regarding the classroom as a mediation setting. On the other hand, there is a higher rating for staff offices (M = 2.19) compared to the scale of where conflicts originate, suggesting that these spaces are perceived as having a clearer conflict-resolution function rather than a conflict-generating one. In contrast, institutional spaces (Dean’s Offices and Deputy Dean’s Offices) maintain the lowest means and the highest positive asymmetries, indicating that conflict management within the administration is perceived as an exceptional and infrequent route by the majority of students.

These descriptive patterns indicate differences in the distribution of perceived conflict across spaces; however, no causal interpretations are derived from these results.

Inferential analyses were then carried out to examine differences in the perception of spaces where conflicts are managed according to gender, thereby addressing the third research question. To analyse differences in the perception of conflict-generating spaces according to gender, tests for differences between means were conducted. Due to the imbalance in group sizes and after verifying the failure of the assumption of homoscedasticity in the most significant factor (Levene’s test, *p* < 0.001), it was decided to apply Welch’s *t*-test (Table 5). The results indicate statistically significant differences only in the ‘Institutional Spaces’ factor (t(Welch)(354.90) = 5.63, *p* < 0.001). In this case, men had a significantly higher mean (M = 8.78; SD = 3.02) than women (M = 7.65; SD = 2.54). To assess the practical relevance of this finding, Hedges’ g was calculated, yielding a value of 0.43 (95% CI [0.29, 0.57]). According to Cohen (1988) criteria, this value represents a moderate effect size, confirming that gender has a substantial influence on the perception of this specific scenario.

Conversely, no statistically significant differences were found in the factors “Workspaces” (t(Welch) = 1.53, *p* = 0.127) nor in “Informal Interaction and Leisure Spaces” (t(Welch) = 1.16, *p* = 0.247), where the effect sizes were trivial (g < 0.111, g = 0.08, respectively).

These data suggest that the gender discrepancy is localised and not a general trend across all the conflict-generating spaces analysed. The findings indicate that gender differences are limited to specific dimensions and are generally associated with small to moderate effect sizes.

With regard to the year of study (Table 5), the analysis of variance revealed that the year of study exerts a statistically significant influence on the perception of conflict in the three dimensions assessed: “Institutional Spaces” (F(3,1227) = 5.20; *p* = 0.001), “Workplaces” (F(3,1247) =3.21; *p* = 0.022) and “Informal Interaction and Leisure Spaces” (F(3,1254) = 3.04; *p* = 0.028). However, the interpretation of these data must be qualified by the size of the effect obtained; the Eta-squared (η^2^) values range from 0.007 to 0.013, representing a small effect that explains barely 1% of the total variance. This disconnect between statistical significance and practical relevance suggests that, whilst there are differences in the means—with Pedagogy standing out in the perception of institutional conflicts (M = 8.44) and Primary Education in workspaces (M = 7.41)—the academic degree does not constitute a determining factor or a robust predictor of conflict. Consequently, these results indicate that the perception of tensions on campus is a cross-cutting and homogeneous phenomenon across the different degree programmes, where the large sample size (N = 1250) has allowed for the detection of minimal differences which, although real in the population, have limited explanatory power in the pedagogical and organisational spheres.

Overall, although some statistically significant differences were identified, the small effect sizes suggest that academic discipline explains only a limited proportion of variance in the perception of conflict.

With regard to the spaces where conflicts are managed (Table 6), a comparison of means was carried out for the gender variable using Welch’s *t*-test, given the heteroscedasticity of the variances (Levene’s test *p* < 0.05) and the imbalance in group sizes. The results revealed statistically significant differences only in the ‘Institutional Settings’ factor (tWelch(349.26) = 2.48, *p* = 0.014), where men scored higher. The effect size for this difference, calculated using Hedges’ g, was 0.191 (95% CI [0.053, 0.331]), indicating a small effect size according to conventional criteria. As for the “Work and Relational Space” factor, Welch’s *t*-test showed that there were no statistically reliable differences (tWelch(376.70) = −1.198; *p* = 0.056). Finally, the “Interaction and Leisure Space” factor also showed no significant differences (tWelch(365.098) = 0.699; *p* = 0.485).

As for the analysis of perceptions of conflict resolution spaces by university year, the results of the one-way ANOVA revealed statistically significant differences only in the “Institutional Spaces” factor (F(3,1231) = 4.51; *p* = 0.004), whilst no significant differences were found for the “Interaction and Leisure Space” (F(3,1238) = 1.80; *p* = 0.145) or for “Work and Relational Space” (F(3,1243) = 1.15; *p* = 0.329). In terms of magnitude, the effect size for “Institutional Spaces” was small (η^2^ = 0.011; 95% CI [0.001, 0.023]), indicating that the degree programme accounts for approximately 1.1% of the variance in this dimension. Multiple comparison tests (Games-Howell) for this factor indicated that Primary Education Teacher Training students exhibit a significantly higher perception (M = 8.93; SD = 3.48) compared to their peers in Early Years Education (M = 8.43; *p* = 0.022), Social Education (M = 8.17; *p* = 0.003) and Pedagogy (M = 8.06; *p* = 0.003). Meanwhile, regarding the ‘Interaction and Leisure’ factor, despite the lack of overall significance in the ANOVA, the post hoc test detected a specific difference between Primary Education (M = 9.12) and Early Years Education (M = 8.65; *p* = 0.023). Finally, the results suggest a consistent perception across all degree programmes regarding the “Work and Interpersonal Space” factor (M(total) = 4.79; SD = 1.24), where significance thresholds were not reached in any of the pairwise comparisons (*p* > 0.05).

Finally, the multivariate analysis of variance (MANOVA) revealed a significant main effect for the gender variable in the overall model (F(418, 790) = 1.19, *p* = 0.019, η^2^ = 0.387), indicating that the students’ gender accounts for a substantial 38.7% of the combined variance in the perception of conflicts. When breaking down this effect using between-subjects tests for the adjusted model, it is observed that this influence is most pronounced in the ‘Institutional Spaces’ dimension (F(16, 790) = 1.84, *p* = 0.023, η^2^ = 0.036), confirming that men and women differ significantly in their assessment of this scenario. Conversely, the academic degree variable did not reach statistical significance in the multivariate model (*p* = 0.990), suggesting that the degree programme undertaken does not significantly influence the perception of conflict. In this regard, the absence of a statistically significant interaction between gender and academic level is confirmed, given that this relationship did not reach the significance thresholds in any of the dimensions analysed (*p* > 0.05). This consistency of results across different programmes suggests that the identified gender gap is a cross-cutting phenomenon and not dependent on the specific curricular context of the students. Therefore, the independence of both variables is assumed, confirming that the differentiated perception between men and women remains constant regardless of the academic year. Furthermore, it is worth noting the emergence of a significant interaction between the Interaction/Leisure and Institutional dimensions based on sex (F (105,790) = 1.27, *p* = 0.042, η^2^ = 0.145), which reinforces the idea that the experience of gender in problem-solving is complex and manifests itself differently depending on the formal or informal nature of the university space analysed.

Although the multivariate model yielded a relatively large effect size in the global test, this result should be interpreted with caution, given the complexity of the model and the smaller effect sizes observed in the individual analyses.

## 5. Discussion

The results of this study provide relevant evidence regarding the spatial dynamics of interpersonal conflicts in the university environment. In line with the conceptualisation of higher education as a relational and symbolic ecosystem (Dong et al., 2023; Wong, 2024), the data indicate that students perceive conflicts to arise primarily in spaces of everyday interaction, such as classrooms, corridors or cafeterias, whilst institutional and administrative spaces (e.g., dean’s offices, counselling services or administration) are perceived as contexts where these occur less frequently. This suggests that conflictual situations tend to arise in environments characterised by frequent interaction, collaborative work and informal communication among students.

These findings must be interpreted in light of the institutional and spatial context in which the study was conducted. Although the sample is limited to students on teacher training programmes at a single Spanish university, the data comes from the three campuses of the University of Granada, an institution with a unique configuration due to its presence in both Europe and Africa. In this sense, the results should not be automatically extrapolated to all education faculties in Spain; however, nor should they be interpreted as a unique, unrepeatable phenomenon. Rather, they can be understood as contextualised evidence with the potential for analytical transfer to other higher education settings characterised by sociocultural diversity, relational complexity and intensive use of shared spaces. From this perspective, the centrality of the classroom, transit spaces and common areas reinforces the idea that the physical and social space of the campus acts as a mediator of university life and the day-to-day management of conflicts (Lozano, 2020; Zenelaga et al., 2025).

From this perspective, spatial dynamics can be understood as the set of physical, social and institutional conditions that shape both the emergence and management of interpersonal conflicts. This approach is consistent with the classical sociocultural perspectives (Vygotsky, 1978) and ecological models of human development (Bronfenbrenner, 1979), which emphasise the role of context in structuring human interaction. The present findings extend these frameworks by showing that specific campus environments function as key settings where tensions are both generated and regulated.

Particularly relevant is the central role of the classroom as a place for both the generation and management of conflicts. Descriptive analyses show that it occurs most frequently in both the emergence and resolution of disputes, with a balanced distribution of responses among participants. These results reinforce the idea that the classroom is a key social microenvironment within campus life, where academic activities, group dynamics and interpersonal interactions converge, consistent with previous studies by Makaremi et al. (2024), Konstantidinis (2024) and Guzmán (2022), who identified the classroom as the space that most accurately reflects the relational reality of the university context.

From a pedagogical perspective, these results highlight the fundamental role of teaching staff in the prevention and management of interpersonal conflicts. Several studies have noted that inclusive teaching practices and the promotion of respectful and participatory learning environments help to strengthen students’ sense of belonging and psychological safety in higher education institutions (Atasanova & Papen, 2025; Brint et al., 2024; Clausen et al., 2025; Misischia, 2022). Consequently, these findings reinforce the need to incorporate specific training in conflict prevention, mediation and dialogue-based resolution strategies into the professional development of university teaching staff (Munuera et al., 2024; Olarte et al., 2025; Zenelaga et al., 2025). However, these interpretations should be approached with caution, as the observed patterns do not necessarily imply strong causal relationships.

In contrast, institutional spaces such as deans’ offices or student support services exhibit a considerably lower incidence of conflictive situations. This result may reflect the hierarchical and regulated nature of these environments, which can act as containment mechanisms that limit the visibility or expression of interpersonal tensions. Dorado et al. (2024) and Tsagris and Pandis (2021) have put forward similar interpretations, highlighting how organisational structures in higher education can influence the visibility and management of conflicts. Furthermore, the low uptake of formal support services identified in this study suggests that students may not fully recognise these resources as accessible channels for conflict resolution. In line with Hernández et al. (2022), this finding underscores the importance of strengthening institutional communication strategies and improving the dissemination of support services, particularly amongst first-year students.

With regard to gender differences, the results indicate that male students report a higher perception of conflict in institutional settings compared to female students. This result differs from previous research, which tends to indicate that women perceive higher levels of insecurity or conflict in university settings (Gargiulo et al., 2020; Huang et al., 2022; Prada-Trigo et al., 2025; Ramos & Pérez, 2025). One possible explanation may lie in differences in conflict coping strategies. Several studies indicate that women tend to adopt more collaborative strategies and to seek social support more frequently, whilst men may employ more avoidant or individualised strategies (Alemany et al., 2024; Rojas et al., 2024b).

The interpretation of these results suggests that formal conflict management is mediated by a differentiated gender perception. Whilst management in work and leisure settings is perceived equally, the institutional structure of the university appears to be experienced differently. The higher score obtained by men on the institutional factor could indicate greater visibility of authority mechanisms for this group, or a greater tendency to formalise complaints. For university institutions, this finding is crucial, as it indicates that services responsible for managing community relations (such as Deans’ Offices and Student Counselling Services) are not perceived neutrally, representing the setting where the gender gap in the perception of university governance becomes most evident. However, these interpretations must be approached with caution, as they could indicate a differential experience of institutional power and the legitimacy to resort to it, which opens up an interesting and relevant line of research on gender and university governance (Jaskulska et al., 2026). Furthermore, the study needs to be supplemented with qualitative data to gain a deeper understanding of the mechanisms underlying these differences.

Finally, the limited effect of academic discipline, despite its statistical significance, reinforces the idea that university conflict is driven more by structural and contextual factors than by academic qualifications. This finding qualifies previous research (Laitinen et al., 2025; Mavor et al., 2025), suggesting that, in contexts where academic qualifications are homogeneous, different identity characteristics may have a lesser impact on the perception and management of conflict.

Overall, the findings should be interpreted with caution. Although statistically significant differences were identified across several dimensions, the effect sizes indicate that these explain only a small proportion of the variance. This suggests that university conflict is a complex and multifactorial phenomenon that cannot be fully explained by isolated individual or academic variables. Therefore, the implications of this study should be understood in terms of general tendencies rather than strong predictive relationships.

Finally, these results reinforce the idea that university space is not a neutral backdrop, but an active component in shaping students’ experiences. Understanding how different environments influence the emergence and management of conflicts is essential for designing institutional policies aimed at promoting coexistence, well-being and meaningful participation in higher education.

## 6. Conclusions

This study contributes to an understanding of the dynamics of interpersonal conflicts in the university context by analysing students’ perceptions of the spaces in which conflicts arise and are managed on campus. The results show that conflict situations are mainly associated with spaces characterised by frequent interpersonal interaction, particularly classrooms, corridors and common areas such as cafeterias. Among these spaces, the classroom emerges as the central context for both the emergence and resolution of conflicts, underscoring its relevance as a key social micro-environment within university life.

This finding is consistent with previous research highlighting the classroom as a central relational space within the university context, where academic activities, collaborative work and interpersonal interactions converge (Guzmán, 2022).

In contrast, institutional spaces, such as deans’ offices or student support services, are perceived as places where conflicts occur less frequently. This finding may reflect the more regulated and hierarchical nature of these environments, which may reduce the visibility of interpersonal tensions or channel them through formal institutional mechanisms.

Dorado et al. (2024) and Tsagris and Pandis (2021) have put forward similar interpretations, in which they highlight that the organisational structures of higher education institutions can influence how conflicts are expressed, perceived and managed.

Furthermore, the results suggest that, although there are some differences in the perception of conflict based on gender and academic discipline, the management of interpersonal disputes tends to be relatively consistent across groups. This pattern supports the idea that conflict management in university settings may be more strongly influenced by contextual and institutional factors than by individual characteristics, as suggested by previous studies on coexistence and relational dynamics in higher education (Dong et al., 2023; Brint et al., 2024).

From a practical perspective, these findings highlight the importance of promoting constructive conflict management strategies in everyday learning environments.

In particular, the results underscore the role of teaching practices and classroom dynamics in fostering respectful interaction, dialogue and collaborative problem-solving among students, as highlighted in previous studies on inclusive and participatory teaching practices in higher education (Atasanova & Papen, 2025; Clausen et al., 2025; Misischia, 2022). Universities should therefore consider strengthening mediation strategies, communication skills and support services aimed at improving interpersonal relations on campus.

The development of institutional policies that promote dialogue-based conflict resolution and a culture of peaceful coexistence can help to improve students’ academic experience, their sense of belonging to the university community and, consequently, their well-being within the university environment.

## 7. Limitations of the Study

Despite the relevance of these findings, certain limitations must be acknowledged. Firstly, the study is based on self-reported perceptions, which may be influenced by subjective interpretations and social desirability bias. In this respect, the data reflect perceptions rather than observed behaviour.

Secondly, the sample comprises students from education degree programmes at a single university; consequently, the results cannot be statistically generalised to all faculties of education or to other university contexts. However, the inclusion of the institution’s three campuses provides internal heterogeneity and allows for a cautious analytical transfer to other higher education settings with complex spatial and social configurations.

Thirdly, the cross-sectional design of the research does not allow for an examination of changes in students’ perceptions of conflict over time. Finally, the quantitative nature of the study limits the scope for exploring in depth the experiences and meanings that students attribute to interpersonal conflicts within university settings, as well as the processes of negotiation and mediation that take place on campus.

Nevertheless, these constraints underscore the pioneering nature of this work and establish a robust baseline for future research aimed at implementing evidence-based coexistence policies on university campuses.

## 8. Future Research

Future studies will address these limitations by incorporating mixed-methods approaches that combine quantitative surveys with qualitative techniques, such as interviews, focus groups or participant observation. This would allow for a more comprehensive understanding of the social processes underlying conflict dynamics in higher education settings.

Furthermore, comparative research across different universities, both in Spain and internationally, would help to identify how institutional cultures and campus spatial configurations influence the perception and management of conflicts. Longitudinal studies would also be particularly valuable for analysing how these perceptions evolve throughout students’ academic careers, from the first year through to postgraduate education, and for assessing the impact of training initiatives aimed at promoting conflict mediation and a culture of peaceful coexistence within universities.

## Figures and Tables

**Table 1:** Questionnaire on Conflict in Universities (CCU).

Section	Name	N Items	Scale Type	Dimensions
B. 1	Personal and academic details	10	Personal and academic identification	
B. 2	Concept of conflict	18	Semantic differential	Beliefs and feelings about conflict
				Conflict resolution
		10	Likert scale	Conflict as a problem and setback
				Conflict as development and progress
B. 3	The most common conflicts	18	Likert scale (1–4) 1 = Never; 2 = Sometimes; 3 = Very often; 4 = Always	Conflicts arising from differences in gender, ethnic background and values
				Vertical conflict
				Conflicts related to the academic environment
				Horizontal conflict
B. 4	Regulatory strategies	13	Likert scale (1–4) 1 = Never; 2 = Sometimes; 3 = Very often; 4 = Always	Positive conflict management strategies
				Conflict avoidance as a form of management
				Mediation as a management technique
				Negative responses to conflict
B. 5	Who to turn to for resolution	17	Likert scale (1–4) 1 = Never; 2 = Sometimes; 3 = Very often; 4 = Always	Management of institutional conflicts
				Conflict management by close colleagues
				Conflict management by close colleagues
B. 6	Areas where conflicts arise	12	Likert scale (1–4) 1 = Never; 2 = Sometimes; 3 = Very often; 4 = Always	Institutional Spaces
				Workplaces
				Spaces for informal interaction and leisure
B. 7	Spaces in which the following aspects are regulated	12	Likert scale (1–4) 1 = Never; 2 = Sometimes; 3 = Very often; 4 = Always	Spaces for interaction and leisure
				Institutional spaces
				Work and social spaces
B. 8	Regulated opening hours	12	Likert scale (1–4) 1 = Never; 2 = Sometimes; 3 = Very often; 4 = Always	

**Table 2:** Spaces where conflicts arise (Block 6) and are resolved (Block 7) at the University.

1. Classes
2. Lecturers’ offices
3. Dean’s offices
4. Deputy Dean’s Offices
5. Student Guidance Services
6. Corridors
7. Canteen
8. Administration
9. Reprographics
10. Off-campus facilities
11. Library
12. Study Rooms

**Table 3:** Descriptive analysis of the spaces where conflicts arise.

Spaces Where Conflicts Arise									
Item	M	SD	Asymmetry	% *	Element	M	SD	Asymmetry	%
Classes	2.76	0.831	0.673	62.2	Canteen	2.04	0.841	0.328	28.5
Offices	1.77	0.710	0.710	12.3	Administration	1.81	0.883	0.836	20.8
Dean’s Office	1.42	0.652	1.48	7.4	Reprographics	1.79	0.804	0.859	16.0
Vice-Deans	1.42	0.648	1.40	4.8	Off-campus	2.11	0.938	0.378	33.7
Orientation	1.52	0.720	1.22	10.8	Library	1.53	0.716	1.30	9.1
Corridors	2.22	0.833	0.271	34.8	Study	1.69	0.777	0.957	13.9

* % Very often or always.

**Table 4:** Descriptive analysis of the spaces where conflicts are managed.

Spaces Where Conflicts Are Managed									
Item	M	SD	Asymmetry	% *	Element	M	SD	Asymmetry	%
Classes	2.64	0.785	−0.010	56.8	Canteen	2.14	0.847	0.282	32.8
Offices	2.19	0.838	0.284	33.8	Administration	1.61	0.770	1.13	12.0
Dean’s Office	1.57	0.770	1.27	11.7	Reprographics	1.55	0.730	1.17	10.6
Vice-Deans	1.53	0.759	1.36	11.1	Off-campus	2.06	0.953	0.442	32.2
Orientation	1.59	0.776	1.17	12.8	Library	1.53	0.739	1.21	11.4
Corridors	2.08	0.818	0.374	28.3	Study	1.63	0.788	1.03	13.9

* % Very often or always.

**Table 5:** Areas where conflicts arise in the university environment according to gender and degree programme.

Spaces	N	Average	SD	t*_Welch_*	*p*	g*_Hedges_*
F1 Institutional Spaces	M = 251	8.78	3.02	5.63	0.000	0.43
	W = 975	7.65	2.54			
F2 Workspaces	M = 257	7.46	2.38	1528	0.127	0.11
	W = 989	7.10	2.32			
F3 Interaction and leisure spaces	M = 257	7.19	1.83	1159	1.23	0.080
	W = 996	7.04	1.88			
University degree being pursued **						
Places of origin	N	Average	SD	f	*p*	Eta squared (η^2^)
F1 Institutional spaces	Ech = 444	7.61	2.47	5206	0.001	0.015
	PE = 402	8.19	2.78			
	SE = 223	7.88	2.90			
	Ped = 162	8.44	3.01			
F2 Workspaces	ECh = 454	7.04	2.27	3.209	0.022	0.001
	PE = 405	7.41	2.36			
	SE = 229	7.02	2.39			
	Ped = 163	6.84	2.29			
F3 Interaction and leisure spaces	Ech = 458	6.93	1.84	3.041	0.028	0.008
	PE = 408	7.20	1.87			
	SE = 230	7.13	1.91			
	Ped = 162	6.74	1.90			

M = Men; W = Women. ** ECh = Early Childhood Education; PE = Primary Education; SE = Social Education; Ped = Pedagogy.

**Table 6:** Spaces where conflicts are managed in the university environment according to gender and degree programme.

Spaces Where Conflicts Are Managed						
Gender *						
Spaces where conflicts are managed	N	M	SD	**t*_Welch_***	***p***	**g*_Hedges_***
F1 Interaction and leisure spaces	253 (M)	9.00	3.21	0.699	0.485	0.052
	985 (W)	8.85	2.90			
F2 Institutional Spaces	251 (M)	8.98	3.52	2.48	0.014	0.191
	980 (W)	8.38	3.05			
F3 Workspaces and relationships	255 (M)	4.65	1.29	−1198	0.056	−0.140
	991 (W)	4.82	1.25			
University degree being pursued **						
Areas where they are managed	N	M	SD	**t**	***p***	Eta squared (η^2^)
F1 Interaction and leisure spaces	ECh = 448	8.43	2.92	4.151	0.006	0.010
	PE = 397	8.87	3.31			
	SE = 227	8.15	3.00			
	Ped = 162	8.05	2.83			
F2 Institutional Spaces	ECh = 452	6.59	2.31	2028	0.108	0.005
	PE = 403	7.01	2,58			
	SE = 229	6.76	2.54			
	Ped = 162	6.77	2.52			
F3 Workspaces and relationships	ECh = 452	6.81	1.72	0.829	0.478	0.002
	PE = 404	6.84	1.81			
	SE = 229	6.88	1.86			
	Ped = 162	7.06	1.80			
	PE = 388	22.70	5.89			
	SE = 224	21.76	5.93			
	Ped = 160	21.85	5.20			

* M: Men; W: Women. ** Ech = Early Childhood Education; PE = Primary Education; SE = Social Education; Ped = Pedagogy.

## Data Availability

The data supporting the findings of this study are available from the corresponding author upon reasonable request. The data are not publicly available due to ethical and confidentiality considerations.
